# Supplementary material for: Clinical assessment of transplacental transfer of maternal SARS-CoV-2 IgG antibodies against spike and nucleocapsid proteins: A chemiluminescence microparticle immunoassay study
Source: PLoS One. 2025 Nov 13;20(11):e0336516. doi: 10.1371/journal.pone.0336516 (PMC12614515; doi:10.1371/journal.pone.0336516)
Supplement: Attachment — Submitted filename: Response to Reviewers.docx [file pone.0336516.s003.docx]

Response to Reviewers

1. **A table showing the vaccination conditions in the study population is required, emphasizing the number of individuals and the type of vaccine with which they were immunized.**

Answer: We acknowledge the importance of this data. Unfortunately, most participants were unable to recall their vaccine type during data collection, and vaccination cards were often unavailable due to the emergency rollout. As national records show, Moderna and Oxford-AstraZeneca were the predominant vaccines administered to pregnant women in Bangladesh during that period. This limitation has been transparently noted in the manuscript.

2. **An interpretation of the presence of specific antibodies to the N protein and the effect these antibodies have on your study is required.**

Answer: We agree with the reviewer’s point and have clarified in the manuscript that anti-N IgG serves as a marker of prior natural infection, given that the N protein is not targeted by COVID-19 vaccines. We have also discussed its limited transplacental transfer and added this reference accordingly.

3. **There is a relationship or effect of antibodies transferred through breast milk on your study.**

Answer: We acknowledge the importance of antibodies transferred through breast milk in providing neonatal immunity. However, our study was specifically designed to assess transplacental IgG transfer, not postnatal transfer via breast milk. As a result, no data regarding antibody levels in breast milk was collected and no consent was taken from the participants regarding this either. Since IgG is the primary antibody transferred across the placenta, and IgA is the predominant immunoglobulin in breast milk, our findings remain focused on placental passive immunity. We have clarified this in the manuscript to avoid overinterpretation of our results.

4. **This study also presents some major limitations, such as the lack of follow-up and the use of convenience sampling, which can introduce bias into the results. Additionally, key variables like the time and type of vaccination and maternal nutritional status were not included. If the authors have access to data that can support their conclusions, it would improve the manuscript significantly.**

Answer: Regarding vaccine type and timing, unfortunately, most participants were unable to recall the specific vaccine received or the exact date of administration, which limited our ability to include these as analytical variables. We also agree that maternal nutritional status could influence antibody generation or transfer, but such data were not systematically collected in our study, and thus were not included in the analysis. Additionally, we employed convenience sampling due to the constraints of hospital-based recruitment during the COVID-19 pandemic, and we recognize the potential for selection bias as a result. Lastly, our study did not include postnatal follow-up, which restricts conclusions about the persistence of maternally derived antibodies in neonates, which was clarified in the limitations already. All of these concerns have been once again clarified in the limitations.

5. **Comparison between A) Maternal Anti-S and Neonatal Anti-S; B) Maternal Anti-N and Neonatal Anti-N; C) Maternal Anti-S and Neonatal Anti-N; D) Maternal Anti-N and Neonatal Anti-S IgG titers (AU/mL) However I can’t find this figure.**

Answer: The figure was originally submitted separately in accordance with the journal’s figure submission guidelines. However, to improve accessibility for reviewers, we have now also included this figure directly within the manuscript (as Figure 1). We hope this will aid in easier reference and enhance the clarity of our findings.

6. **The authors assume the functionality of the antibodies based solely on their presence; no functional assays were performed. Please elaborate on and disclose the limitations of the findings to avoid overinterpretation of the results.**

   Answer: We fully agree that inferring protective immunity based solely on antibody presence could lead to overinterpretation. To address this, we have revised the Discussion section to clearly state this limitation and acknowledge that functional assays would be required to confirm antibody efficacy.
7. **The use of anti-N IgG is problematic since the study did not separate the volunteers by vaccine type. Considering this humoral response in the context of vaccination without mentioning the influence of natural infection could mislead the findings. This limitation also extends to the correlation with PTR, as these antibodies might have been produced in different contexts.**

   Answer: We acknowledge that anti-N IgG antibodies are produced only following natural SARS-CoV-2 infection, and not in response to vaccination with currently authorized vaccines, which are spike-based. As most participants could not recall their vaccine type, and we could not verify this retrospectively, it was not possible to stratify the cohort based on vaccination status or type. We also agree that the presence of anti-N IgG may reflect prior infection rather than vaccine induced immunity, and we have revised the Discussion section to reflect this interpretation more cautiously.
8. **No control group was included. A comparison between the PTR of vaccinated and non-vaccinated women could help determine whether the antibody levels are vaccine-related.**
   Answer: It’s true that the lack of a control group of unvaccinated women limits our ability to directly compare placental transfer ratios (PTRs) between vaccinated and non-vaccinated individuals. However, this study was conducted during a period when COVID-19 vaccination was nationally mandated for all pregnant women, and as such, recruiting a well-matched unvaccinated control group was not feasible.
9. **There is no justification provided for the use of Spearman correlation.**

   Answer: Thank you for pointing this out. We have clarified in the revised manuscript that Spearman’s correlation was chosen because not all variables (for example: PTRs, maternal and neonatal antibody titers) followed a normal distribution, as assessed by the Shapiro-Wilk test. Additionally, we have also added the justification for Mann-Whitney U test, and the statistical analysis section has been edited accordingly.
10. **Additional:
    A.** We have submitted the raw data containing Excel file for possibly replicating the results of this study.
    **B.** Reviewer #2 said we have mentioned in our study that the pregnant women included in our study knew what type of vaccines they were administered with. We believe it’s an honest mistake as no such information was mentioned. Moreover, we have mentioned the lack of type of vaccination data as a limitation also.
